# The Effects of Classroom Interventions on Off-Task and Disruptive Classroom Behavior in Children with Symptoms of Attention-Deficit/Hyperactivity Disorder: A Meta-Analytic Review

**DOI:** 10.1371/journal.pone.0148841

**Published:** 2016-02-17

**Authors:** Geraldina F. Gaastra, Yvonne Groen, Lara Tucha, Oliver Tucha

**Affiliations:** Department of Clinical and Developmental Neuropsychology, University of Groningen, Groningen, The Netherlands; Philipps University Marburg, GERMANY

## Abstract

Children with attention-deficit/hyperactivity disorder (ADHD) often exhibit problem behavior in class, which teachers often struggle to manage due to a lack of knowledge and skills to use classroom management strategies. The aim of this meta-analytic review was to determine the effectiveness of several types of classroom interventions (antecedent-based, consequence-based, self-regulation, combined) that can be applied by teachers in order to decrease off-task and disruptive classroom behavior in children with symptoms of ADHD. A second aim was to identify potential moderators (classroom setting, type of measure, students’ age, gender, intelligence, and medication use). Finally, it was qualitatively explored whether the identified classroom interventions also directly or indirectly affected behavioral and academic outcomes of classmates. Separate meta-analyses were performed on standardized mean differences (SMDs) for 24 within-subjects design (WSD) and 76 single-subject design (SSD) studies. Results showed that classroom interventions reduce off-task and disruptive classroom behavior in children with symptoms of ADHD (WSDs: *M*_SMD_ = 0.92; SSDs: *M*_SMD_ = 3.08), with largest effects for consequence-based (WSDs: *M*_SMD_ = 1.82) and self-regulation interventions (SSDs: *M*_SMD_ = 3.61). Larger effects were obtained in general education classrooms than in other classroom settings. No reliable conclusions could be formulated about moderating effects of type of measure and students’ age, gender, intelligence, and medication use, mainly because of power problems. Finally, classroom interventions appeared to also benefit classmates’ behavioral and academic outcomes.

## Introduction

Attention-deficit/hyperactivity disorder (ADHD) is a neurodevelopmental disorder characterized by inattention and/or hyperactivity-impulsivity [1]. Approximately 5 to 7% of all children meet diagnostic criteria for ADHD [2,3], implying that on average every classroom will contain a child with ADHD. Within the classroom, children with ADHD are more inattentive (off-task) and disruptive than typically developing peers [4,5]. They often struggle to sustain attention to tasks and instructions, frequently talk to classmates at inappropriate times, and may call out and leave their seat without permission [6]. As a consequence, children with ADHD are at risk of academic difficulties, including underachievement, retaining grade, special educational placement, and suspension or drop-out from school [7–10]. Moreover, as ADHD related behaviors may disturb the learning process of classmates [6] and may elicit maladaptive behavior of both classmates and teacher [11], overall classroom functioning may decrease, both academically and socially. Campbell, Endman, and Bernfeld [12] suggested that the presence of a child with ADHD in the classroom leads to more negative interaction between teacher and students.

As teachers may be confronted daily with one or more children with ADHD in their classroom, it is important that they have confidence managing these children. General education teachers, irrespectively of their age and years of teaching experience, perceive children with ADHD as more stressful to teach than other children [13]. They report that teaching children with ADHD causes a disruption of the teaching process, a loss of satisfaction from teaching, self-doubt and increased need for support [13,14]. Teacher factors have a considerable impact on the achievement and behavioral outcomes of children with ADHD [15], but often teachers also seem to lack knowledge and skills to develop and implement effective classroom interventions [16]. Frequently simple management techniques are used, which can be implemented classwide and are not time-consuming [16–18], whereas interventions that are based on an analysis of the function of an individual child’s behavior (function-based interventions) have been proven to be more effective than non-function based interventions [19]. As many children with ADHD attend general education classrooms [10], it is important to assist teachers in their management of these children. Providing teachers with effective tools may benefit children with ADHD as well as their classmates, but moreover, may improve confidence and well-being of teachers themselves.

The problem behaviors of students with ADHD in the classroom may require treatment. The most common treatment for children with ADHD is stimulant medication [6,7]. Although pharmacological interventions enhance on-task behavior and academic achievement in children with ADHD [20], pharmacological interventions are limited by several factors, including possible side effects, lack of evidence of long-term effects, and compliance problems [21–25]. Furthermore, medical treatment may not normalize behavior and cognition in children with ADHD [26,27]. Because of these significant limitations, there is a need for non-pharmacological interventions, including school-based interventions.

The effectiveness of school-based interventions for ADHD was previously examined by a number of meta-analytic studies, indicating that school-based interventions improve behavioral and academic outcomes of children with ADHD [28–31]. DuPaul and Eckert [28] and DuPaul et al. [29] performed extensive meta-analyses of published and unpublished studies on school-based interventions for children with ADHD. The first study included 63 studies covering a period of 24 years (1971−1995) and the follow-up study included 60 studies covering a successive period of 14 years (1996−2010). Both studies indicated that school-based interventions improve behavior in children with ADHD but that effects on academic outcomes are smaller and less robust. The studies also compared different types of interventions, including academic, contingency management, and cognitive-behavioral interventions. The effects of intervention type were inconsistent between the two studies and depended on the experimental design applied (i.e., between-subjects, within-subjects, or single-subject design) and the outcomes collected (i.e., behavioral or academic outcomes). Inconsistent results were also found for moderating effects of school setting and educational placement, which may be caused by the small number of studies included for some moderator categories.

Two other meta-analytic studies describe a more narrow span of research. Purdie et al. [30] examined the effectiveness of different types of interventions, including school-based interventions, on several types of outcomes (behavioral, cognitive, social, and personal/emotional outcomes) of individuals with ADHD. The meta-analysis included eight studies on school-based interventions covering a period of eight years (1990−1998). The results showed small positive effects for school-based interventions on all types of outcomes. For cognitive outcomes, the effects were larger for school-based interventions than for other types of interventions (pharmacological, non-school-based psychological, parent training, multimodal interventions). Another meta-analysis specifically focused on studies that implemented self-regulation interventions for children with ADHD in school settings, and included 16 studies covering a period of 29 years (1974−2003) [31]. Positive effects of self-regulation interventions were found for on-task behavior, inappropriate behavior, and academic accuracy and productivity.

### The present study

The present study provides a meta-analytic review of published studies on classroom interventions for ADHD covering a period of 33 years of research (1970−October 2013). The primary aim was to determine the effectiveness of several types of classroom interventions (antecedent-based, consequence-based, self-regulation, combined interventions) that can be applied by teachers in order to decrease off-task and disruptive classroom behavior in children with symptoms of ADHD. A second objective was to identify potential moderators (classroom setting, type of measure, students’ age, gender, intelligence, and medication use). As previous meta-analyses on this topic did not investigate such a wide time frame, potential moderators could be more robustly analyzed. Furthermore, it was qualitatively explored whether the identified classroom interventions also affected the behavioral and academic outcomes of classmates, which has not been addressed before. It was hypothesized that classroom interventions could have positive effects on classmates; either because of indirect effects i.e., less classroom disturbance by children with symptoms of ADHD, or because of direct effects i.e., improvement of classmates’ behavior because they also benefit from the intervention. This study will provide information on evidence-based classroom management of children with ADHD behavior, and the outcomes may be of relevance and use in the education of teachers.

## Method

There existed no protocol for this meta-analytic review. The guidelines for Preferred Reporting Items for Systematic Reviews and Meta-analyses (PRISMA) were followed (see S1 Checklist).

### Inclusion and exclusion criteria

In order to be included in this meta-analytic review, studies had to meet the following inclusion criteria.

1. The study was published in English in an academic journal. Initially, the aim was to include unpublished studies but due to limited resources and time, it was decided to restrict the meta-analytic review to published studies.
2. Participants attended grades 1 through 12 (if grade was not reported, age 6 to 17 years) and had ADHD, ADD, attention deficits, or hyperactive-impulsive deficits. Furthermore, participants had an IQ of 70 or above (if IQ was reported). No restrictions were made regarding comorbid conditions and medication use. For studies examining classroom interventions for different medication dosages, placebo conditions were included in the analysis.
3. In order to be able to generalize the results to the natural classroom and the general education teacher (having limited resources and limited advanced skills), the following requirements were defined. The intervention had to be implemented in the classroom by the teacher (or an experimenter who could be easily replaced by a teacher) and required no parental involvement. For example, interventions incorporating parent training or school-home notes with parent-delivered consequences were not included. The intervention had to take place in a classroom context, i.e., in the presence of a teacher and some peers of the child with symptoms of ADHD.
4. The intervention could be classified into one of the following categories of classroom interventions or into a combination of these categories:
  - Antecedent-based intervention: An intervention that manipulates antecedent conditions, such as the environment, task, or instruction (e.g., seating, music, tutoring, choice making, computer-assisted instruction).
  - Consequence-based intervention: An intervention that uses reinforcement and punishment to alter the frequency of target behavior (e.g., praise, reprimands, prizes, privileges, response-cost).
  - Self-regulation intervention: An intervention aimed at the development of self-control and problem-solving skills to regulate cognition and behavior (e.g., self-instruction, self-monitoring, self-reinforcement).
5. The outcome measures were either teacher ratings or direct observations of off-task behavior (e.g., not attending to task or teacher, looking around), disruptive behavior (e.g., disturbing classmates, playing with objects, out of seat), and ADHD behavior (e.g., teacher rating on an ADHD rating scale) in the classroom. Measures of on-task behavior (e.g., attending to task or teacher, face directed towards work sheet) and appropriate behavior (e.g., absence of oppositional behaviors, compliance with requests) were also included, because these are by definition mutually exclusive to off-task, disruptive, and ADHD behavior. That is, a child cannot be on-task while at the same time being off-task. A reduction of off-task, disruptive, or ADHD behavior and an increase in on-task or appropriate behavior was regarded as a positive effect of the intervention. Outcome measures that were obtained outside the classroom (e.g., playground) were excluded.
6. The study could be classified into one of the following categories of experimental design categories:
  - Between-subjects group design: A design that uses an intervention group and a non-intervention control group.
  - Within-subjects group design (WSD): A design that applies the same intervention on each participant and assesses outcomes on at least two occasions.
  - Single-subject design (SSD): A design that documents changes in behavior for an individual participant during intervention phases and non-intervention control phases. Studies using a control group but with assessments on at least two occasions, were considered to be WSD studies as this would increase the number of studies included in the same meta-analysis. In case both individual and group data (e.g., means across participants) were provided, the study was categorized as a group design study.
7. Sufficient data were provided to compute effect sizes. If studies included participants both with and without symptoms of ADHD, the results had to allow disaggregation for these participants. In case of insufficient data, the authors of the relevant studies were contacted.

### Search procedure

A systematic literature search was conducted to identify studies for inclusion in the meta-analytic review. First of all, electronic database searches in PsycINFO, ERIC, and Web of Science were performed until the date of October 8, 2013. A combination of search terms was used to describe participants (*ADHD*, *ADD*, *attention deficit*, *hyperactivity*, or *hyperkinetic*), interventions (*classroom*, *school*, *education*, or *academic*, and *treatment*, *intervention*, *training*, *strategies*, *therapy*, or *program*), and outcome measures (*classroom*, *school*, *academic*, *on-task*, *off-task*, or *disruptive*, and *functioning* or *behavior*). The search was restricted to the English language and in PsycINFO additionally limited to school-aged children (6 to 11 years) and adolescents (12 to 17 years). Furthermore, reference lists of relevant literature reviews and of studies included in the present meta-analytic review were checked for additional studies. All records identified by the electronic and manual searches were screened based on title and abstract. The full text of the remaining articles was used to determine eligibility for inclusion.

### Coding procedure and moderating variables

Each study meeting inclusion criteria was systematically coded on several variables by the first author. Variables that were examined as potential moderators included intervention type, classroom setting, type of measure, and characteristics of participants receiving the intervention, including age, gender, intelligence, and medication use. The categories *antecedent-based*, *consequence-based*, *self-regulation*, and *combined* were used to classify intervention type. Classroom setting was defined as the classroom in which the intervention was implemented and coded as (inclusive) *general education* or *other* (e.g., special education, self-contained, resource, remedial, experimental, laboratory, hospital classroom). If an intervention was implemented in both the general education classroom and another classroom setting, it was classified as *other*. Type of measure was coded as *teacher ratings*, *direct observations*, or *both*. The mean or range of the age or grade of participants was used to create age categories for *children* (age 6 to 11 years; otherwise grade 1 through 5) and *adolescents* (age 12 to 17 years; otherwise grade 6 through 12). Gender was defined as the percentage of boys of the study samples and classified as *less than 20% boys*, *20 to 80% boys*, or *more than 80% boys*. Mean IQ of participants was used to allocate samples to the IQ categories *less than 90*, *90 to 110*, or *above 110*. Finally, medication use was defined as the percentage of participants on medication during the study and was classified as *less than 20% medicated*, *20 to 80% medicated*, or *more than 80% medicated*.

Study quality of all studies included was assessed by means of the method developed by Reichow and colleagues [32,33] for the evaluation of the methodological quality (i.e. rigor) of group as well as SSD studies on evidence-based practices. Both primary and secondary quality indicators were rated conform specific operational definitions. Primary quality indicators are critical for evaluating the validity of studies and are rated as *high quality*, *acceptable quality*, and *unacceptable quality*. Secondary quality indicators are important though not necessary elements for the validity of studies, and are rated as *evidence* or *no evidence*. Based on the ratings of primary and secondary quality indicators, an overall study quality rating was calculated (*strong*, *adequate*, or *weak*).

A random subset of 30 studies was coded by an independent second rater. Interrater reliability statistics were computed (see S1 Table). Raters agreed between 80 and 100% (*M* = 96%, κ = .94) on general study information (e.g., number of participants). For WSD studies, interrater reliability ranged from 70 to 100% agreement (*M* = 92%, κ = .85, κ_w_ = .91) for primary quality indicators and from 90 to 100% agreement (*M* = 96%, κ = .89) for secondary quality indicators. For SSD studies, raters agreed between 55 and 100% (*M* = 83%, κ = .76, κ_w_ = .89) on primary quality indicators and 90 and 100% (*M* = 98%, κ = .95) on secondary quality indicators. Overall agreement between raters on overall study quality across study designs was 80% (κ = .56, κ_w_ = .71). Coding disagreements were resolved through discussion with the co-authors.

### Statistical analyses

Statistical procedures were conducted using statistical software (*IBM SPSS statistics 22*). To maintain independence between effect sizes, studies were only allowed to contribute a maximum of one effect size for each intervention category. For studies providing multiple outcomes, mean effect sizes across outcomes were computed. For SSD studies including more than one participant, mean effect sizes across participants were computed. A positive effect size indicated an improvement in behavior.

Effect sizes used for this meta-analytic review were defined as standardized mean differences (SMDs). For WSD studies, SMDs were computed as described by Becker [34]. This method allows for combining studies with and without control group in the same meta-analysis. A description of the method used for computation of effect sizes for WSDs is provided in S1 Text. For SSD studies, there is no consensus on appropriate methods for calculation of effect sizes [35]. To allow for comparison between the present study and previous meta-analyses on school interventions for ADHD, SMDs were calculated. SSD studies either reported data as descriptive statistics (means and standard deviations) or within graphs. In the latter case, data points were extracted from graphs by measuring them with the help of a ruler. The first baseline and last intervention phase were used for computation of effect sizes. Each of these phases had to consist of at least three data points to demonstrate existence or lack of an effect [35]. SMDs were computed by dividing the difference between the means of the intervention and baseline phases by the pooled standard deviation [36,37]. These were then corrected for small numbers of data points [38]. Because exact expressions for effect size variances of SSDs have not been derived and are formally not justified [35,39], standard errors and consequently statistical significance tests were not conducted for SSD studies. The distribution of effect sizes was examined separately for WSD and SSD studies. Effect sizes deviating more than two standard deviations from the mean of all effect sizes (across the studies of a particular experimental design) were recoded (i.e., Winsorized) to less extreme values. This reduced the impact of extremely large or small effect sizes on the outcomes.

Separate meta-analyses were conducted for studies employing WSDs and SSDs because effect size estimators in WSD and SSD studies are fundamentally different [39]. For the analysis of the WSD studies, macros were used that were created by Lipsey and Wilson [40]. The mean weighted effect size was computed using a random effects model [41]. Moderator analyses were conducted using mixed effects models, assuming that identifiable study characteristics act as moderator variables but that some unmeasured random effect remains [40]. Each effect size was weighted by its inverse variance. Heterogeneity was assessed by performing homogeneity tests and calculating the I^2^ value. I^2^ reports the proportion of total variation across studies that is due to heterogeneity rather than chance [42]. Values in the order of 25%, 50%, and 75% may be considered as low, moderate, and high, respectively. To detect publication or related bias, funnel plot asymmetry was tested using a regression method [43]. Furthermore, the fail-safe N was computed [44] in order to determine the number of studies with an effect size of zero that would be necessary to reduce the mean effect size to criterion levels of 0.20 (small effect) and 0.50 (medium effect) [45].

To determine the direct and indirect intervention effects on the classmates of participants with symptoms of ADHD, effect sizes using the same formulas as for participants with symptoms of ADHD were calculated. In case insufficient data were provided, the means of baseline and intervention phases were used to compute the percentage of change in behavior or academic performance. Because only a small number of studies provided information on classmates, the results are discussed descriptively without performing a meta-analysis.

## Results

**Fig 1:**
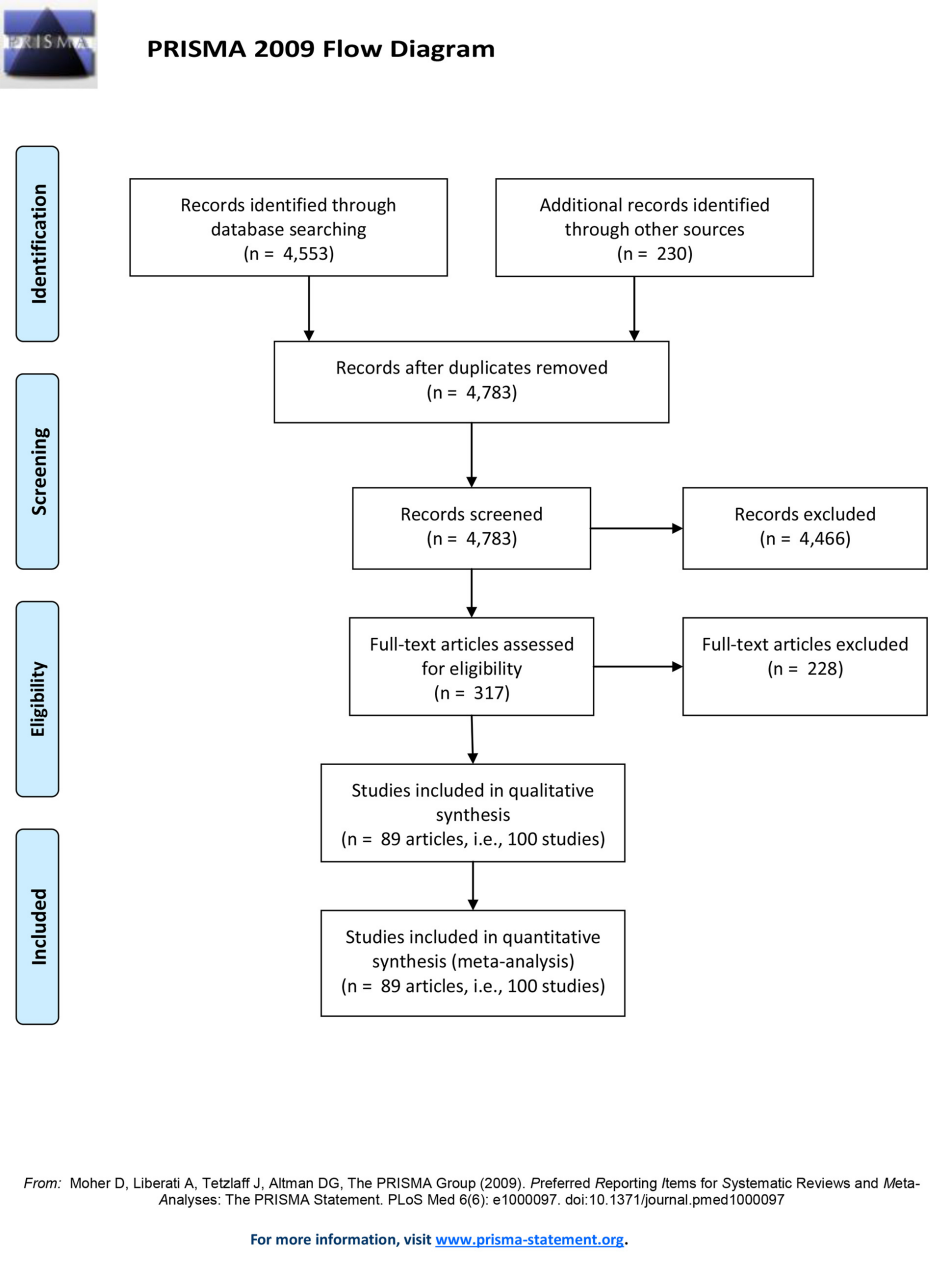
Flow Diagram of Literature Search.

An overview of the literature search is provided in Fig 1. A total of 4,553 records were identified through electronic databases and an additional 230 records were identified by the manual searches. Screening of the titles and abstracts of these records resulted in 317 articles. Inspection of the full-texts of these 317 articles resulted in the exclusion of 228 articles that failed to meet inclusion criterion. An article could provide more than one effect size (study) for analysis if the article reported on multiple interventions from different categories of interventions or reported on multiple experiments. Finally, a total of 89 articles meeting inclusion criteria were considered in the present meta-analytic review, yielding to 100 studies. The list of studies included in the meta-analytic review is provided in S2 Text.

### Study characteristics

Table 1 summarizes the major characteristics of studies included in this meta-analytic review. For each decade in the period 1970 to October 2013, there is a steady increase in the number of newly published studies on classroom interventions for children with symptoms of ADHD. The majority of studies employed SSDs (76%), whereas none of the studies solely used a between-subjects design. Of the 24 WSD studies, one third also included a control group consisting of participants with symptoms of ADHD who did not receive an intervention. A small number of studies provided information about the direct (8%) and indirect effects (3%) of classroom interventions on classmates of participants with symptoms of ADHD. Combined interventions (10%) were less often implemented than other types of interventions, whereas antecedent-based, consequence-based, and self-regulation interventions were approximately equally often implemented. Interventions were as often implemented in general education classrooms (46%) as in other classroom settings (47%). Most measures consisted of direct observations (93%), whereas only a small number of studies gathered teacher ratings (4%) or both types of outcome measures (3%). In total, 627 participants (WSDs: *n* = 471; SSDs: *n* = 156) were included in the meta-analytic review. The study samples varied between 1 and 65 participants, with the majority of studies including 10 or less participants (87%). Most studies included children (84%) and predominantly boys (74%). IQ was assessed in 27% of the studies, with most of these studies (18%) reporting a mean IQ in the average range (90−110). Medication use varied between studies, with 27% of studies not providing information about medication status of participants.

**Table 1:** Characteristics of Studies Included in the Meta-Analytic Review.

Characteristic	*k*	Characteristic	*k*
Year of publication		Number of participants	
≤ 1980	9	1−10	87
1981–1990	19	11−20	6
1991–2000	24	21−30	1
2001–2010	30	≥ 31	6
≥ 2011	18	Age	
Experimental design		Children	84
Between-subjects	0	Adolescents	16
Within-subjects	24	Gender	
Control group	8	≤ 19% boys	6
No control group	16	20−80% boys	19
Single-subject	76	≥ 81% boys	74
Examination classmates		Not provided	1
No	89	IQ	
Yes	11	≤ 89	5
Type of measure		90−110	18
Teacher ratings	4	≥ 111	4
Observations	93	Not provided	73
Both	3	Medication use	
Intervention type		≤ 19% medicated	33
Antecedent-based	26	20−80% medicated	12
Consequence-based	33	≥ 81% medicated	28
Self-regulation	31	Not provided	27
Combined	10	Study quality	
Classroom setting		Strong	4
General education	46	Adequate	43
Other	47	Weak	53
Not provided	7		

A frequencies table of the primary, secondary, and overall quality ratings is provided in S2 Table. Regarding primary quality indicators, most WSD studies were rated as high of quality on the indicators ‘independent variable’ (i.e., description of the intervention) (92% high quality), ‘dependent variable’ (i.e. description of the outcome measure) (83% high quality), and ‘link to research question’ (100% high quality) but were rated as unacceptable of quality on ‘comparison condition’ (i.e., definition of a control group) (67% unacceptable quality). Regarding secondary quality indicators, most WSD studies showed evidence of ‘interobserver agreement’ (63% evidence) and ‘social validity’ (83% evidence) but no evidence for the other secondary quality indicators (i.e., ‘random assignment’ (8% evidence), ‘blind raters’ (25% evidence), ‘fidelity’ (33% evidence), ‘attrition’ (21% evidence), ‘generalization or maintenance’ (4% evidence), ‘effect size’ (8% evidence)). Overall, the majority of WSD studies (83%) obtained a weak rating of study quality. SSD studies were generally rated as high of quality on ‘independent variable’ (100% high quality) and ‘dependent variable’ (86% high quality) but varied in quality on the other primary quality indicators. Most SSD studies reported good ‘interobserver agreement’ (90% evidence) and showed evidence of ‘social validity’ (95% evidence). However, the majority of SSD studies showed no evidence of the other secondary quality indicators (ranging from 71 to 96% no evidence). Overall, half of the SSD studies (54%) had an adequate study quality and 43% were rated as weak.

### Within-subjects design studies

For WSD studies, one outlier effect size value (*SMD* = 3.51) was identified for a study implementing a consequence-based intervention. This value was Winsorized to a less extreme value of 3.00. A summary of the characteristics of each WSD study included in the meta-analysis is provided in S3 Table. Summary statistics for the moderator analyses are shown in Table 2. For WSD studies, IQ was not included in the moderator analyses because only eight of these studies reported IQ of participants.

**Table 2:** Summary Statistics for Moderator Analyses for Within-Subjects and Single-Subject Design Studies.

	Within-subjects designs				Single-subject designs	
	*k* = 24, *n* = 471				*k* = 76, *n* = 156	
			95% CI			
Category	*k*	*M*_SMD_	*LL*	*UL*	*k*	*M*_SMD_
Intervention type						
Antecedent-based	9	0.31	0.06	0.55	17	2.65
Consequence-based	8	1.82	1.39	2.24	25	2.47
Self-regulation	4	0.56	0.02	1.11	27	3.61
Combined	3	0.58	0.07	1.08	7	2.59
Classroom setting						
General education	10	1.30	0.82	1.78	36	3.58
Other	14	0.64	0.26	1.02	33	2.41
Type of measure						
Teacher ratings	4	0.85	0.07	1.64	−	−
Direct observations	17	1.01	0.59	1.44	76	3.08
Both	3	0.82	−0.16	1.80	−	−
Age						
Children	22	1.02	0.67	1.38	62	3.00
Adolescents	2	0.26	−0.77	1.29	14	3.39
Gender						
≤ 19% boys	0	−	−	−	6	2.63
20−80% boys	8	1.12	0.49	1.75	11	3.68
≥ 81% boys	15	0.88	0.45	1.32	59	2.87
Medication use						
≤ 19% medicated	12	0.91	0.40	1.41	21	2.34
20−80% medicated	3	0.98	0.05	1.91	9	3.16
≥ 81% medicated	2	0.46	−0.72	1.65	26	3.22

**Fig 2:**
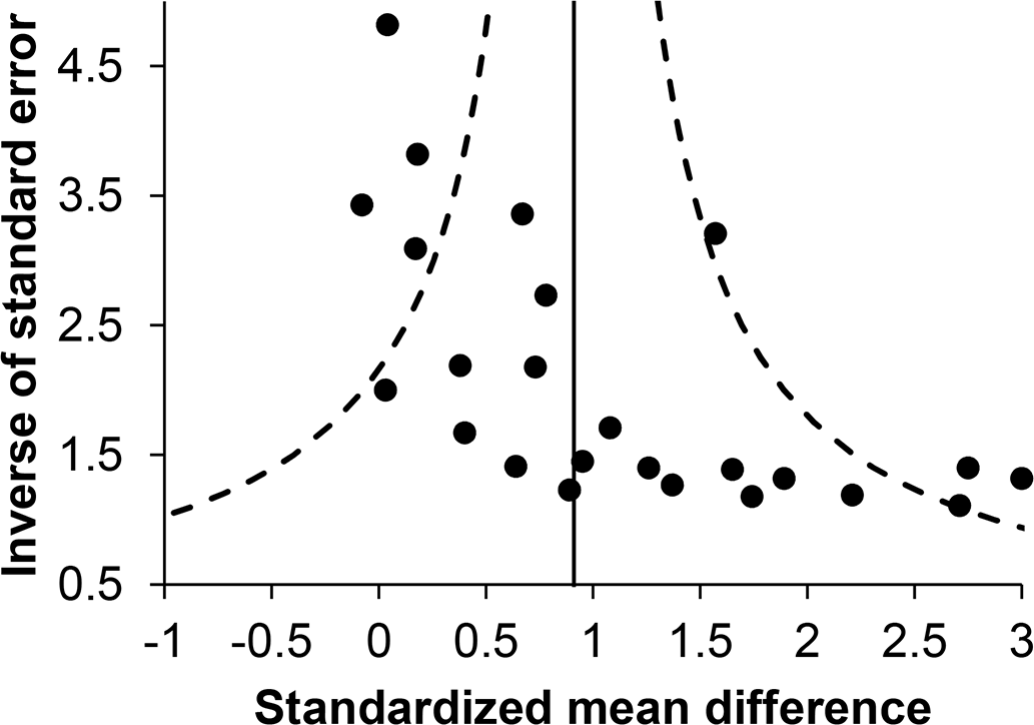
Funnel Plot of Within-Subjects Design Studies. A funnel plot showing the effect sizes of within-subjects design studies as a function of the inverse of their standard error. The vertical line indicates the weighted mean effect size (*M*_SMD_ = 0.92) and the dashed lines represent the 95% confidence limits around this mean.

Effect sizes for WSD studies ranged from −0.08 to 3.00 (Winsorized value) with a median of 0.92. The mean weighted effect size was 0.92 and reached significance (95% CI [0.59, 1.25]). Effect sizes were significantly heterogeneous (*Q*_T_ [23] = 66.80, *p* < .001; *I*^2^ = 66%), indicating potential moderators. A significant effect for intervention type was found (*Q*_B_ [3] = 36.77, *p* < .001), with consequence-based interventions producing larger effects than antecedent-based, self-regulation, and combined interventions. Effect sizes also differed significantly for classroom setting (*Q*_B_ [1] = 4.43, *p* = .035), with larger effects for interventions implemented in general education classrooms than for interventions implemented in other classroom settings. No significant effect was found for type of measure (*Q*_B_ = [2] = 0.20, *p* = .904), age (*Q*_B_ = [1] = 1.88, *p* = .170), gender (*Q*_B_ [1] = 0.37, *p* = .543), and medication use (*Q*_B_ [2] = 0.53, *p* = .769). To detect publication and related bias, a funnel plot was created (Fig 2). The funnel plot showed significant asymmetry (*p* < .001), which seemed to be due to missing of smaller studies showing no or small beneficial effects. Fail-safe N analyses showed that 86.4 and 20.2 studies with effect sizes of zero would be necessary to reduce the mean effect size to 0.20 and 0.50, respectively.

### Single-subject design studies

For SSD studies, two outlier effect size values were identified for studies implementing a consequence-based (*SMD* = 12.43) and self-regulation intervention (*SMD* = 8.17). These values were Winsorized to a less extreme value of 7.00. A summary of the characteristics of each SSD study included in the meta-analysis is provided in S4 Table. Summary statistics for moderator examination are provided in Table 2. Type of measure and IQ were not included in the moderator examination of SSD studies because all effect sizes were obtained for direct observations and only 19 studies reported IQ of participants. No significance tests could be performed as exact expressions for effect size variances for SSDs have not been derived and are formally not justified.

Effect sizes for SSD studies ranged from 0.42 to 7.00 (Winsorized value) with a median of 2.63. The mean weighted effect size was 3.08. Regarding intervention type, effect sizes were largest for self-regulation interventions (*M*_SMD_ = 3.61) and smallest for consequence-based interventions (*M*_SMD_ = 2.47). Regarding classroom setting, largest effects were obtained in general education classrooms (*M*_SMD_ = 3.58) and smallest in other classroom settings (*M*_SMD_ = 2.41). The examination of age as a potential moderator, resulted in a mean effect size of 3.00 for studies including children and a mean effect size of 3.39 for studies conducted in adolescents. Regarding gender, the largest effect sizes were found for mixed samples of boys and girls (*M*_SMD_ = 3.68). Finally, medication use was examined as moderator. Studies including a high proportion of participants on medication achieved largest effect sizes (*M*_SMD_ = 3.22), whereas studies with a low rate of medicated participants showed smallest effect sizes (*M*_SMD_ = 2.34).

### Direct effects on classmates

Four WSD and four SSD studies provided information that could be useful for the assessment of direct effects of classroom interventions on classmates of children with symptoms of ADHD (see Table 3). For all four WSD studies applying antecedent-based interventions, effect sizes for behavioral outcomes of classmates were positive, ranging from 0.21 to 1.97 [46–49]. One of these studies also included academic performance measures of classmates and revealed an effect size of 0.64 [46]. Positive effects on classmates were also found for all four SSD studies. Two SSD studies applying an antecedent-based [50] and a self-regulation intervention [51] produced effect sizes of 1.96 and 2.53, respectively. For the other two SSD studies applying a consequence-based and a self-regulation intervention, mean class’ disruptive behavior decreased on average with 52% and 36% respectively during intervention phases as compared to baseline phases [52].

**Table 3:** Summary of Studies Examining Direct Effects of Classroom Interventions on Classmates of Children With Symptoms of ADHD.

Study: reference number^a^	*n*	Intervention	Intervention type	Outcome	Effect
Within-subjects designs					
[46]	9	Classwide peer-tutoring	Antecedent-based	Active on-task, passive on-task, off-task, fidgeting behavior	*SMD* = 0.25, 95% CI [−0.97, 1.47]
	10	Classwide peer-tutoring	Antecedent-based	Academic performance	*SMD* = 0.64, 95% CI [−0.28, 1.56]
[47]	76	Stability balls	Antecedent-based	ADHD teacher ratings	*SMD* = 0.28, 95% CI [−0.04, 0.60]
[48]	8	Formal classroom	Antecedent-based	Hyperactive behavior	*SMD* = 1.97, 95% CI [0.60, 3.34]
[49]	26	Music at background	Antecedent-based	On-task behavior	*SMD* = 0.21, 95% CI [−0.34, 0.76]
Single-subject designs					
[51]	4	Self-management + peer-monitoring within a group contingency	Self-regulation	Uncontrolled verbalizations	*SMD* = 2.53
[52]^1^	Class	Teacher-administered classwide reinforcement	Consequence-based	Disruptive behavior	52% decrease
[52]^2^	Class	Classwide self-management procedures	Self-regulation	Disruptive behavior	36% decrease
[50]	3	Recess	Antecedent-based	Inappropriate behavior	*SMD* = 1.96

^a^ Superscript numbers are added to references that yielded more than one study.

### Indirect effects on classmates

Three studies, all SSD studies, provided information showing indirect effects of classroom interventions on classmates of children with symptoms of ADHD (see Table 4). Positive indirect effects on behavioral outcomes of classmates were found for two out of three studies. An effect size of 1.46 was obtained for a study implementing a self-regulation intervention [53]. Two other studies applying a combined [54] and a self-regulation intervention [55] used data of different classmates and showed a behavioral improvement (34% decrease in off-task behavior) and deterioration (2% decrease in on-task behavior) respectively during intervention phases compared to baseline phases. The latter study also included classmate academic performance measures and observed an increase of 6% in academic performance during intervention phases compared to baseline phases.

**Table 4:** Summary of Studies Examining Indirect Effects of Classroom Interventions on Classmates of Children With Symptoms of ADHD.

Study: Reference number	*n*	Intervention	Intervention type	Outcome	Effect
[54]	Different classmates	Skill training + differential reinforcement + self-monitoring	Combined	Off-task behavior	34% decrease
[55]	Different classmates	Self-management procedures	Self-regulation	On-task behavior	2% decrease
	Different classmates	Self-management procedures	Self-regulation	Academic productivity	6% increase
[53]	3	Self-monitoring	Self-regulation	On-task behavior	*SMD* = 1.46

## Discussion

The primary aim of this meta-analytic review was to determine the effectiveness of several types of classroom interventions that can be applied by teachers in order to decrease off-task and disruptive classroom behavior in children with symptoms of ADHD. The results indicate that classroom interventions reduce off-task and disruptive classroom behavior in children with symptoms of ADHD, which is in accordance with previous meta-analyses [28–31]. Large effects were found for WSD studies (*M*_*SMD*_ = 0.92). Positive effects were also found for SSD studies (*M*_SMD_ = 3.08) but interpreting the absolute magnitude of these effects is difficult as statistical guidelines for such interpretation are lacking. It should be noted that effect sizes for WSDs and SSDs cannot be directly compared to each other as they represent different units of measurement. The obtained effect sizes were somewhat larger than those found in other meta-analyses of studies on school-based interventions for ADHD [28–31], which may have several causes. First, unlike the present study, DuPaul and Eckert [28] and DuPaul et al. [29] included unpublished studies. Furthermore, Purdie et al. [30] only included eight school-based studies and Reid et al. [31] specifically focused on self-regulation interventions, which resulted in a more selective overview. Finally, there were differences in the exact method of computation of effect sizes, the outcomes used, and the interventions examined.

WSD studies indicate that consequence-based interventions (*M*_SMD_ = 1.82) are more effective in reducing off-task and disruptive classroom behavior in children with symptoms of ADHD than antecedent-based (*M*_SMD_ = 0.31), self-regulation (*M*_SMD_ = 0.56), and combined interventions (*M*_SMD_ = 0.58). However, SSD studies showed largest effects for self-regulation interventions (*M*_SMD_ = 3.61) and smallest effects for consequence-based interventions (*M*_SMD_ = 2.47). The discrepancy in results between the two types of research designs may be the consequence of differences between the characteristics of participants (e.g., medication use) or the specific interventions that were implemented. Based on the present study, it can be concluded that the different classroom interventions performed had (small to large) positive effects on off-task and disruptive classroom behavior in children with symptoms of ADHD, with consequence-based and self-regulation interventions showing the strongest effects.

The results indicate that interventions implemented in general education classrooms (WSDs: *M*_SMD_ = 1.30; SSDs: *M*_SMD_ = 3.58) lead to a larger reduction in off-task and disruptive classroom behavior in children with symptoms of ADHD than interventions implemented in other classroom settings (WSDs: *M*_SMD_ = 0.64; SSDs: *M*_SMD_ = 2.41). This difference may be explained by different populations allocated to or different treatments performed in these settings. For example, the more positive effects in general education classrooms compared to other classroom settings could be explained by the fact that children with less severe symptoms of ADHD and/or less comorbidities are generally included in general education classrooms. These children may benefit more from classroom interventions than children with more severe symptoms of ADHD and/or more comorbidities, who tend to be included in special education classrooms. Furthermore, in special education classrooms there may be less room for improvement compared to general education classrooms because behavioral programs are already in place in special education classrooms.

Unfortunately, no reliable conclusions can be drawn on the influence of the type of measure used because most studies reported direct observations and only few effect sizes could be computed for teacher ratings (because either they were not reported or too few data points were available). Other studies examining the relationship between teacher ratings and observational data indicate that these two measurements of behavior are weakly to strongly correlated [56,57], suggesting that classroom interventions would not only improve direct observations but also improve teacher ratings. It is important that classroom interventions do not only improve behavior as measured objectively by direct observations but also improve behavior subjectively as perceived by teacher, because teachers whose efforts are rewarded may become more confident and motivated to both educate children with symptoms of ADHD and change their classroom management in favor of these children [58].

Finally, there was no clear evidence that the age, gender, and medication use of participants influenced the results. For SSD studies, intervention effects seemed to be similar for children and adolescents, and largest for studies including a mix of boys and girls. SSD studies did show a trend for a positive influence of medication on the effectiveness of classroom interventions for individuals with ADHD. However, this could not be tested statistically. For WSD studies, no moderating effects of age, gender, and medication use were found. This, however, may have been caused by a low statistical power due to the limited availability of studies in some of the categories. Intelligence as a moderator was not examined in the present study because most studies did not provide information on participants’ cognitive level. For future studies on school-based interventions, it is therefore clearly recommended to include cognitive measures as well as both genders and age groups.

The small number of studies that provided information on the effects of classroom interventions on classmates of children with symptoms of ADHD indicates positive effects on overall classroom functioning. Classmates who received the same intervention as participants with symptoms of ADHD as well as classmates who did not receive any intervention themselves, showed an improvement in behavioral and academic outcomes. This implies that classroom interventions for children with symptoms of ADHD have both direct effects on classmates, i.e., improvement of classmates’ behavior because they also benefit from the intervention, and indirect effects on classmates, i.e., profit from less classroom disturbance by children with symptoms of ADHD. Although positive effects on classmates were found for all types of classroom interventions, most studies reported on direct effects of antecedent-based interventions and indirect effects of self-regulation interventions.

### Limitations

There are several factors that limit the conclusions of this meta-analytic review. First, this meta-analytic review was restricted to studies published in academic journals, which most likely has resulted in an upward bias in effect sizes. However, it is unlikely that small or negative effects would be obtained if unpublished studies would have been included, as the fail-safe N analyses for WSD studies indicate that as many as 86 studies with effect sizes of zero would be necessary to reduce the effect size to small. Furthermore, there was a trend for the smaller studies to show larger treatment effects than the larger studies, which may be due to differences in methodological quality. Examination of study quality indicates that many group and SSD studies are weak in methodological quality. For example, most group studies did not include a control group (67% unacceptable quality) and a substantial number of SSD studies had problems with demonstrating experimental control (17% unacceptable quality). This limits the interpretation of the results. Moreover, most studies employed SSDs, for which exact expressions of effect size variances have not been derived [35,39]. Therefore, moderator examination of SSD studies was descriptive and consequently did not allow for firm conclusions. Additionally, no statistical guidelines exist for the interpretation of effect sizes for SSD studies, which also limits the interpretation of the present findings. For WSD studies, some moderator effects may have been missed because of the low number of studies performed with regard to the effects of some moderators. Also, potential interactions between moderating variables could not be examined.

Another limitation is that potential moderators had to be analyzed using subgroup analyses instead of meta-regression analysis because the data were not normally distributed and therefore violated the assumptions for regression analysis. The use of categorical instead of continuous variables may have resulted in a loss of precision and power. Furthermore, the use of rather broad categories of classroom interventions did not allow conclusions about specific classroom interventions within these categories. Also, the outcomes applied within the studies were considerable heterogeneous. For example, some studies used a broad definition of off-task behavior (e.g., ‘not on-task’), whereas other studies defined specific types of disruptive behavior (e.g., ‘uncontrolled verbalizations’).

Finally, this meta-analytic review was restricted regarding the age and gender of participants, and type of measure. The results are most representative for boys in the age of 6 to 11 years, as only a minority of studies reported about samples including females and/or adolescents. Furthermore, the results were most often obtained from direct observation measures, reflecting objective behavior and not subjective behavior as perceived by the teacher. However, these two measurements of behavior have been found to be correlated [56,57], suggesting that the results of this meta-analytic review are not only applicable to objective behavior of students but may also be generalized to teachers’ experiences of students’ behavior.

### Future research

The present study highlights several areas of recommended future research. First, studies on classroom interventions for ADHD have mainly focused on boys and elementary school children. As girls and adolescents with symptoms of ADHD may respond differently, there is a particular need for research on these samples. Additional factors influencing the effectiveness of classroom interventions for children with symptoms of ADHD should be further examined, because the current meta-analytic review showed considerably heterogeneous effect sizes that could not be fully explained by the investigated moderators. For example, child factors other than age and gender (e.g., cognitive dysfunctions, medication use), teacher factors (e.g., teaching experience, personality), and potential interactions between the different factors should also be taken into account. Finally, most studies evaluating classroom interventions for ADHD have employed SSDs or group designs of weak methodological quality. There is a need for higher quality studies, especially large-scale studies using randomized controlled designs, that allow for more reliable and firm conclusions.

### Implications for practice

The findings of the current study are promising because they indicate that teachers can effectively implement classroom interventions to reduce off-task and disruptive classroom behavior in children with symptoms of ADHD. All types of interventions examined appeared to be effective but strongest effects were obtained for consequence-based (for WSD studies) and self-regulation interventions (for SSD studies), suggesting that teachers should consider such types of interventions in particular. The appropriateness of a specific type of intervention depends on the characteristics of the child as well as the function and meaning of his or her ADHD-related behavior [19]. Therefore, it is important that teachers consider which interventions are effective for an individual child (in contemplation with a professional such as a school psychologist or internal supervisor).

The results also indicate that classroom interventions are most effective in general education classrooms, which is promising as many children with ADHD attend such classrooms [10]. Furthermore, children with symptoms of ADHD who are on medication also benefit from classroom interventions. Therefore, teachers should not be reluctant to implement classroom interventions for children with symptoms of ADHD who already receive medical treatment for their problems, because current data do not exclude that classroom interventions provide additional improvement to medical treatment. Finally, teachers do not have to be concerned about a potential negative impact of above classroom interventions on overall classroom functioning, as the current results denote positive effects, both direct and indirect, of classroom interventions on classmates of children with symptoms of ADHD.

Because teachers often seem to lack knowledge and skills to develop and implement effective classroom interventions for children with ADHD [16], it is recommended that classroom management training is offered to teachers. Such training would not only provide teachers with effective tools for classroom management of children with symptoms of ADHD but may also improve their confidence and well-being. Consequently, such training is likely to be beneficial to children with symptoms of ADHD, their classmates, as well as their teachers.

### Conclusion

This meta-analytic review indicates that classroom interventions reduce off-task and disruptive classroom behavior in children with symptoms of ADHD. WSD studies showed that consequence-based interventions are more effective than antecedent-based, self-regulation, and combined interventions. However, SSD studies showed largest effects for self-regulation interventions. Larger effects were obtained for children with symptoms of ADHD in general education classrooms than for those in other classroom settings. No reliable conclusions can be formulated about moderating effects of type of measure, and student’s age, gender, intelligence, and medication use. Finally, the study also indicates positive direct and indirect effects of these classroom interventions on classmates’ behavioral and academic outcomes. The results of this study may be used for educating and training teachers in dealing with children with symptoms of ADHD.

## Supporting Information

S1 PRISMA Checklist PRISMA Checklist. (DOC)

S1 Table Interrater reliability. (DOCX)

S2 Table Study quality. (DOCX)

S3 Table Characteristics of Within-Subjects Design Studies Included in the Meta-Analytic Review. (DOCX)

S4 Table Characteristics of Single-Subject Design Studies Included in the Meta-Analytic Review. (DOCX)

S1 Text Computation of Effect Sizes. (DOCX)

S2 Text List of Studies Included in the Meta-Analytic Review. (DOCX)
