# Supplementary material for: The Effects of Classroom Interventions on Off-Task and Disruptive Classroom Behavior in Children with Symptoms of Attention-Deficit/Hyperactivity Disorder: A Meta-Analytic Review
Source: PLoS One. 2016 Feb 17;11(2):e0148841. doi: 10.1371/journal.pone.0148841 (PMC4757442; doi:10.1371/journal.pone.0148841)
Supplement: S1 Table — (DOCX) [file pone.0148841.s002.docx]

**S1 Table. Interrater reliability.**

Interrater reliability statistics for general study information.

| Study characteristic | % agreement | κ^a^ |
| --- | --- | --- |
| Year of publication | 100 | 1.00 |
| Experimental design | 100 | 1.00 |
| Control group | 100 | 1.00 |
| Examination classmates | 100 | 1.00 |
| Type of measure | 100 | 1.00 |
| Intervention type | 93 | .91 |
| Classroom setting | 97 | .94 |
| Number of participants | 100 | 1.00 |
| Age | 100 | 1.00 |
| Gender | 97 | .92 |
| IQ | 90 | .81 |
| Medication use | 80 | .71 |

Interrater reliability statistics for primary quality indicators, secondary quality indicators, and overall study quality for within-subjects design studies.

|  | % agreement | κ^a^ | κ_w_^b^ |
| --- | --- | --- | --- |
| Primary quality indicators |  |  |  |
| Participant characteristics | 90 | .78 | .80 |
| Independent variable | 100 | 1.00 | 1.00 |
| Comparison condition | 100 | 1.00 | 1.00 |
| Dependent variable | 90 | .78 | .80 |
| Link to research question | 100 | ^c^ | ^c^ |
| Statistical analyses | 70 | .55 | .85 |
| Secondary quality indicators |  |  |  |
| Random assignment | 90 | .62 |  |
| Interobserver agreement | 90 | .78 |  |
| Blind raters | 100 | 1.00 |  |
| Fidelity | 100 | 1.00 |  |
| Attrition | 90 | .74 |  |
| Generalization or maintenance | 100 | 1.00 |  |
| Effect size | 100 | 1.00 |  |
| Social validity | 100 | 1.00 |  |
| Overall study quality | 90 | .76 | .89 |

Interrater reliability statistics for primary quality indicators, secondary quality indicators, and overall study quality for single-subject design studies.

|  | % agreement | κ^a^ | κ_w_^b^ |
| --- | --- | --- | --- |
| Primary quality indicators |  |  |  |
| Participant characteristics | 90 | .83 | .90 |
| Independent variable | 100 | ^c^ | ^c^ |
| Baseline | 80 | .76 | .83 |
| Dependent variable | 100 | 1.00 | 1.00 |
| Visual analysis | 75 | .63 | .87 |
| Experimental control | 55 | .36 | .76 |
| Secondary quality indicators |  |  |  |
| Interobserver agreement | 90 | .69 |  |
| Kappa | 100 | 1,00 |  |
| Blind raters | 100 | ^c^ |  |
| Fidelity | 100 | 1.00 |  |
| Generalization or maintenance | 100 | 1.00 |  |
| Social validity | 100 | ^c^ |  |
| Overall study quality | 75 | .47 | .54 |

^a^Siegel S, Castellan NJ. *Nonparametric statistics for the behavioral sciences (2nd ed.).* New York, NY, England: Mcgraw-Hill Book Company; 1988.

^b^Cohen J. Weighted kappa: Nominal scale agreement provision for scaled disagreement or partial credit. *Psychol Bull*. 1968;70(4):213-220.

^c^Kappa could not be computed due to lack of variability in scores.
