# Supplementary material for: The Effects of Classroom Interventions on Off-Task and Disruptive Classroom Behavior in Children with Symptoms of Attention-Deficit/Hyperactivity Disorder: A Meta-Analytic Review
Source: PLoS One. 2016 Feb 17;11(2):e0148841. doi: 10.1371/journal.pone.0148841 (PMC4757442; doi:10.1371/journal.pone.0148841)
Supplement: S2 Table — (DOCX) [file pone.0148841.s003.docx]

**S2 Table. Study quality.**

Frequencies (percentage of studies) of scores on primary quality indicators, secondary quality indicators, and overall study quality for within-subjects design studies.

| Primary quality indicators | High quality (%) | Acceptable quality (%) | Unacceptable quality (%) |
| --- | --- | --- | --- |
| Participant characteristics | 58.3 | 8.3 | 33.3 |
| Independent variable | 91.7 | 8.3 | - |
| Comparison condition | 20.8 | 12.5 | 66.7 |
| Dependent variable | 83.3 | 16.7 | - |
| Link to research question | 100 | - | - |
| Statistical analyses | 41.7 | 20.8 | 37.5 |
| Secondary quality indicators | Evidence (%) | No evidence (%) |  |
| Random assignment | 8.3 | 91.7 |  |
| Interobserver agreement | 62.5 | 37.5 |  |
| Blind raters | 25.0 | 75.0 |  |
| Fidelity | 33.3 | 66.7 |  |
| Attrition | 20.8 | 79.2 |  |
| Generalization or maintenance | 4.2 | 95.8 |  |
| Effect size | 8.3 | 91.7 |  |
| Social validity | 83.3 | 16.7 |  |
|  | Strong (%) | Adequate (%) | Weak (%) |
| Overall study quality | 8.3 | 8.3 | 83.3 |

Frequencies (percentage of studies) of scores on primary quality indicators, secondary quality indicators, and overall study quality for single-subject design studies.

| Primary quality indicators | High quality (%) | Acceptable quality (%) | Unacceptable quality (%) |
| --- | --- | --- | --- |
| Participant characteristics | 40.8 | 55.3 | 3.9 |
| Independent variable | 100.0 | - | - |
| Baseline | 56.6 | 32.9 | 10.5 |
| Dependent variable | 85.5 | 14.5 | - |
| Visual analysis | 59.2 | 25.0 | 15.8 |
| Experimental control | 61.8 | 21.1 | 17.1 |
| Secondary quality indicators | Evidence (%) | No evidence (%) |  |
| Interobserver agreement | 89.5 | 10.5 |  |
| Kappa | 5.3 | 94.7 |  |
| Blind raters | 3.9 | 96.1 |  |
| Fidelity | 28.9 | 71.1 |  |
| Generalization or maintenance | 14.5 | 85.5 |  |
| Social validity | 94.7 | 5.3 |  |
|  | Strong (%) | Adequate (%) | Weak (%) |
| Overall study quality | 2.6 | 53.9 | 43.4 |
