# Supplementary material for: The Effects of Classroom Interventions on Off-Task and Disruptive Classroom Behavior in Children with Symptoms of Attention-Deficit/Hyperactivity Disorder: A Meta-Analytic Review
Source: PLoS One. 2016 Feb 17;11(2):e0148841. doi: 10.1371/journal.pone.0148841 (PMC4757442; doi:10.1371/journal.pone.0148841)
Supplement: S3 Table — (DOCX) [file pone.0148841.s004.docx]

**S3 Table. Characteristics of Within-Subjects Design Studies Included in the Meta-Analytic Review.**

| Study: Authors, year^a^ | *n*_i_ (*n*_c_) | % boys | Age | % med | Setting | Intervention | Intervention type | Measure | *SMD*, 95% CI |
| --- | --- | --- | --- | --- | --- | --- | --- | --- | --- |
| Barkley, Copeland, & Sivage, 1980 | 6 | 100 | Children | - | Other | Self-control procedures | Self-regulation | Direct observations | 1.74  [0.08, 3.40] |
| Bloomquist, August, & Ostrander, 1991 | 12 (13) | ±69 | Children | 0 | General education | Teacher training | Combined | Both | 0.03  [−0.94, 1.01] |
| Bowers, Clement, Fantuzzo, & Sorensen, 1985^1^ | 6 | 100 | Children | - | Other | Teacher-administered reinforcement | Consequence-based | Direct observations | 0.64  [−0.75, 2.02] |
| Bowers, Clement, Fantuzzo, & Sorensen, 1985^2^ | 6 | 100 | Children | - | Other | Self-reinforcement | Self-regulation | Direct observations | 1.37  [−0.18, 2.91] |
| DuPaul, Ervin, Hook, & McGoey, 1998 | 18 | 83 | Children | 0 | General education | Classwide peer-tutoring | Antecedent-based | Direct observations | 1.08  [−0.06, 2.23] |
| Eastman & Rasbury, 1981 | 6 (5) | - | Children | - | General education | Self-instruction training | Self-regulation | Direct observations | 0.89  [−0.70, 2.49] |
| Evans et al., 1995 | 14 | 93 | Adolescents | - | Other | Notetaking | Antecedent-based | Direct observations | 0.38  [−0.52, 1.27] |
| Fedewa & Erwin, 2011 | 8 | 75 | Children | 0 | General education | Stability balls | Antecedent-based | Both | 2.21  [0.55, 3.86] |
| Flynn & Rapoport, 1976 | 10 (13) | 100 | Children | 90 | General education | Open classroom | Antecedent-based | Teacher ratings | 0.95  [−0.41, 2.30] |
| Guderjahn, Gold, Stadler, & Gawrilow, 2013 | 57 | 88 | Adolescents | 82 | Other | Self-regulation strategies | Self-regulation | Teacher ratings | 0.17  [−0.47, 0.80] |
| Hart, Massetti, Fabiano, Pariseau, & Pelham, 2011 | 33 | 76 | Children | 64 | Other | Small-group instruction | Antecedent-based | Direct observations | 0.67  [0.08, 1.25] |
| Jacob, O'Leary, & Rosenblad, 1978 | 8 | 88 | Children | 0 | Other | Formal classroom | Antecedent-based | Direct observations | 0.40  [−0.78, 1.58] |
| Jurbergs, Palcic, & Kelley, 2010 | 13 (16) | 85 | Children | 23 | General education | Daily report cards without parent delivered consequences | Consequence-based | Direct observations | 3.00 (Winsorized)  [1.52, 4.48] |
| Kapalka, 2005 | 45 (41) | 100 | Children | - | General education | Effective commands | Consequence-based | Teacher ratings | 1.57  [0.96, 2.18] |
| Miranda, Jarque, & Rosel, 2006 | 17 (16) | 82 | Children | 0 | General education | Teacher training | Combined | Teacher ratings | 0.73  [−0.17, 1.63] |
| Miranda, Presentacion, & Soriano, 2002 | 29 (21) | 90 | Children | 0 | General education | Teacher training | Combined | Both | 0.78  [0.07, 1.50] |
| Palcic, Jurbergs, & Kelley, 2009 | 13 (16) | 54 | Children | 15 | General education | Daily report cards without parent delivered consequences | Consequence-based | Direct observations | 2.75  [1.35, 4.15] |
| Pariseau, Fabiano, Massetti, Hart, & Pelham, 2010 | 33 | 76 | Children | 64 | Other | Extended time | Antecedent-based | Direct observations | −0.08  [−0.65, 0.49] |
| Pelham et al., 2011^1^ | 41 | 100 | Children | 0 | Other | Music at background | Antecedent-based | Direct observations | 0.18  [−0.33, 0.69] |
| Pelham et al., 2011^2^ | 65 | 100 | Children | 0 | Other | Music at background | Antecedent-based | Direct observations | 0.04  [−0.36, 0.45] |
| Pfiffner, Rosén, & O'Leary, 1985 | 8 | 63 | Children | - | Other | Negative consequences | Consequence-based | Direct observations | 2.71  [0.95, 4.48] |
| Rosén, O'Leary, Joyce, Conway, & Pfiffner, 1984^1^ | 8 | 100 | Children | 0 | Other | Prudent negative consequences | Consequence-based | Direct observations | 1.89  [0.41, 3.38] |
| Rosén, O'Leary, Joyce, Conway, & Pfiffner, 1984^2^ | 8 | 63 | Children | 0 | Other | Prudent negative consequences | Consequence-based | Direct observations | 1.65  [0.24, 3.06] |
| Rosén, O'Leary, Joyce, Conway, & Pfiffner, 1984^3^ | 7 | 57 | Children | 0 | Other | Prudent negative consequences | Consequence-based | Direct observations | 1.26  [−0.14, 2.66] |

*Note. n*_i_ = number of participants in intervention group; *n*_c_ = number of participants in control group; med = medicated.

^a^Superscript numbers are added to references that yielded more than one study.
