# Supplementary material for: The Effects of Classroom Interventions on Off-Task and Disruptive Classroom Behavior in Children with Symptoms of Attention-Deficit/Hyperactivity Disorder: A Meta-Analytic Review
Source: PLoS One. 2016 Feb 17;11(2):e0148841. doi: 10.1371/journal.pone.0148841 (PMC4757442; doi:10.1371/journal.pone.0148841)
Supplement: S4 Table — (DOCX) [file pone.0148841.s005.docx]

**S4 Table. Characteristics of Single-Subject Design Studies Included in the Meta-Analytic Review.**

| Study: Authors, year^a^ | *n* | % boys | Age | % med | Setting | Intervention | Intervention type | *SMD* |
| --- | --- | --- | --- | --- | --- | --- | --- | --- |
| Abramowitz, Eckstrand, O'Leary, & Dulcan, 1992 | 3 | 100 | Children | 0 | Other | Immediate reprimands | Consequence-based | 2.69 |
| Alter, Wyrick, Brown, & Lingo, 2008^1^ | 1 | 100 | Children | 100 | General education | Problem-solving strategy training | Self-regulation | 2.32 |
| Alter, Wyrick, Brown, & Lingo, 2008^2^ | 1 | 100 | Children | 100 | General education | Problem-solving strategy training + token economy | Combined | 5.88 |
| Anhalt, McNeil, & Bahl, 1998 | 1 | 0 | Children | - | General education | ADHD Classroom Kit | Consequence-based | 1.82 |
| Ardoin & Martens, 2004 | 4 | 100 | Children | - | General education | Self-evaluation with accuracy training | Self-regulation | 1.07 |
| Banda & Sokolosky, 2012 | 1 | 100 | Children | 100 | General education | Noncontingent teacher attention | Antecedent-based | 2.79 |
| Barry & Messer, 2003 | 5 | 100 | Adolescents | 100 | General education | Self-management procedures | Self-regulation | 3.03 |
| Broussard & Northup, 1995 | 1 | 100 | Children | 100 | General education | Differential reinforcement of other behavior | Consequence-based | 3.79 |
| Broussard & Northup, 1997 | 2 | 100 | Children | 100 | General education | Differential reinforcement of other behavior | Consequence-based | 2.91 |
| Burley & Waller, 2005 | 1 | 100 | Adolescents | 0 | General education | Reinforcement of low rates of disruptive behavior | Consequence-based | 3.07 |
| Cameron & Robinson, 1980 | 3 | 67 | Children | 0 | Other | Cognitive training | Self-regulation | 1.17 |
| Campbell & Anderson, 2011 | 2 | 100 | Children | 100 | Other | Teacher feedback | Consequence-based | 0.51 |
| Carter & Horner, 2009 | 1 | 100 | Children | 0 | General education | Function-based behavioral support plan | Consequence-based | 0.79 |
| Christie, Hiss, & Lozanoff, 1984 | 3 | 100 | Children | 0 | General education | Self-recording | Self-regulation | 3.09 |
| Coleman, 1970 | 2 | 100 | Children | - | General education | Conditioning technique | Consequence-based | 2.63 |
| Davies & Witte, 2000 | 4 | 50 | Children | 100 | General education | Self-management & peer-monitoring within a group contingency | Self-regulation | 4.56 |
| DiGangi, Maag, & Rutherford, 1991 | 2 | 0 | Children | - | General education | Self-management procedures | Self-regulation | 2.13 |
| DiGennaro, Martens, & McIntyre, 2005 | 1 | 0 | Adolescents | - | General education | Reinforcement-based intervention plan | Consequence-based | 1.17 |
| Ducharme & Harris, 2005 | 1 | 100 | Children | 0 | Other | Errorless embedding | Antecedent-based | 2.16 |
| Dunlap et al., 1994 | 1 | 100 | Children | 100 | Other | Choice making | Antecedent-based | 1.73 |
| DuPaul, Guevremont, & Barkley, 1992 | 2 | 100 | Children | 50 | Other | Attention Training System with directed rehearsal | Consequence-based | 1.85 |
| DuPaul & Henningson, 1993 | 1 | 100 | Children | 0 | General education | Classwide peer tutoring | Antecedent-based | 3.28 |
| Ervin, DuPaul, Kern, & Friman, 1998^1^ | 1 | 100 | Adolescents | 100 | - | Brainstorming & computer use | Antecedent-based | 2.48 |
| Ervin, DuPaul, Kern, & Friman, 1998^2^ | 1 | 100 | Adolescents | 100 | - | Self-evaluation procedures | Self-regulation | 3.34 |
| Fabiano & Pelham, 2003 | 1 | 100 | Children | 0 | General education | Modified reward & feedback | Consequence-based | 1.94 |
| Germer et al., 2011 | 1 | 100 | Children | 0 | General education | Antecedent + reinforcement adjustments | Combined | 3.39 |
| Gordon et al., 1991 | 6 | 50 | Children | 0 | Other | Attention Training System | Consequence-based | 1.12 |
| Graham-Day, Gardner, & Hsin, 2010 | 3 | 67 | Adolescents | 67 | Other | Self-monitoring with reinforcement | Self-regulation | 2.45 |
| Gureasko-Moore, DuPaul, & White, 2007 | 6 | 100 | Adolescents | 33 | General education | Self-management procedures | Self-regulation | 4.27 |
| Gureasko-Moore, DuPaul, & White, 2006 | 3 | 100 | Adolescents | 100 | General education | Self-management procedures | Self-regulation | 3.62 |
| Hallahan, Lloyd, Kneedler, & Marshall, 1982 | 1 | 100 | Children | - | Other | Self-monitoring | Self-regulation | 4.86 |
| Hallahan, Lloyd, Kosiewicz, Kauffman, & Graves, 1979 | 1 | 100 | Children | - | Other | Self-monitoring | Self-regulation | 2.63 |
| Harris, 1986 | 4 | 50 | Children | - | Other | Self-monitoring | Self-regulation | 2.77 |
| Harris, Friedlander, Saddler, Frizzelle, & Graham, 2005 | 6 | 83 | Children | 100 | General education | Self-monitoring | Self-regulation | 2.55 |
| Hoff & Ervin, 2013^1^ | 2 | 100 | Children | 50 | General education | Teacher-administered classwide reinforcement | Consequence-based | 1.08 |
| Hoff & Ervin, 2013^2^ | 2 | 100 | Children | 50 | General education | Classwide self-management procedures | Self-regulation | 1.42 |
| Hoff, Ervin, & Friman., 2005 | 1 | 100 | Adolescents | 100 | General education | Preferred peer far & more-preferred materials | Antecedent-based | 2.46 |
| Horn, Chatoor, & Conners, 1983 | 1 | 100 | Children | 0 | Other | Self-control procedures | Self-regulation | 0.53 |
| Iskander & Rosales, 2013^1^ | 2 | 100 | Children | 0 | Other | Social Story | Self-regulation | 0.44 |
| Iskander & Rosales, 2013^2^ | 2 | 100 | Children | 0 | Other | Social Story + differential reinforcement of zero rates of behavior | Combined | 1.26 |
| Kern, Delaney, Clarke, Dunlap, & Childs, 2001 | 1 | 100 | Children | 100 | Other | Choice of medium | Antecedent-based | 1.10 |
| Kubany, Weiss, & Sloggett, 1971 | 1 | 100 | Children | - | General education | Good behavior clock | Consequence-based | 4.91 |
| Lee, Sugai, & Horner, 1999 | 1 | 100 | Children | 100 | Other | Academic instruction | Antecedent-based | 0.72 |
| Lloyd, Hallahan, Kosiewicz, & Kneedler, 1982 | 1 | 100 | Children | - | Other | Self-recording | Self-regulation | 2.61 |
| Lo & Cartledge, 2006 | 2 | 100 | Children | 50 | Other | Skill training + differential reinforcement + self-monitoring | Combined | 0.80 |
| Locke & Fuchs, 1995 | 3 | 100 | Children | 67 | Other | Peer-mediated instruction | Antecedent | 1.95 |
| Maag, Rutherford, & DiGangi, 1992 | 6 | 67 | Children | - | General education | Self-management procedures | Self-regulation | 4.58 |
| Majeika et al., 2011 | 1 | 100 | Adolescents | 100 | General education | Antecedent + reinforcement adjustments | Combined | 2.63 |
| Mathes & Bender, 1997 | 3 | 100 | Children | 100 | Other | Self-monitoring | Self-regulation | 6.56 |
| Mautone, DuPaul, & Jitendra, 2005 | 2 | 100 | Children | 0 | Other | Computer-assisted instruction | Antecedent-based | 3.51 |
| Nolan & Filter, 2012 | 1 | 100 | Children | - | - | Noncontingent reinforcement + response cost | Consequence-based | 5.00 |
| Northup, Broussard, Jones, & George, 1995 | 3 | 67 | Children | 0 | Other | Differential reinforcement of other behavior | Consequence-based | 4.24 |
| Ota & DuPaul, 2002 | 3 | 100 | Children | 100 | Other | Computer-assisted instruction | Antecedent-based | 1.93 |
| Pang & Zhang, 2011 | 3 | 67 | Adolescents | - | - | Reading intervention | Antecedent-based | 3.02 |
| Paniagua, Morrison, & Black, 1990 | 1 | 100 | Children | 0 | Other | Reinforcement of promise-do correspondence | Consequence-based | 1.19 |
| Paniagua, Pumariega, & Black, 1988^1^ | 2 | 100 | Children | 0 | Other | Reinforcement of corresponding reports/fulfillment of promises | Consequence-based | 1.24 |
| Paniagua, Pumariega, & Black, 1988^2^ | 1 | 100 | Children | 0 | Other | Reinforcement set up on promise | Consequence-based | 2.83 |
| Powell & Nelson, 1997 | 1 | 100 | Children | 100 | General education | Choice making | Antecedent-based | 2.14 |
| Price, Martella, Marchand-Martella, & Cleanthous, 2002 | 1 | 100 | Children | 100 | General education | Immediate feedback through FM headset | Consequence-based | 1.26 |
| Rafferty, Arroyo, Ginnane, & Wilczynski, 2011 | 3 | 67 | Children | 67 | General education | Self-monitoring | Self-regulation | 5.70 |
| Rapport, Murphy, & Bailey, 1982 | 2 | 100 | Children | 0 | General education | Response cost | Consequence-based | 4.28 |
| Rapport et al., 1980^1^ | 1 | 100 | Children | 0 | - | Response cost | Consequence-based | 7.00  (Winsorized) |
| Rapport et al., 1980^2^ | 1 | 0 | Children | 0 | - | Response cost | Consequence-based | 3.38 |
| Ridgway, Northup, Pellegrin, LaRue, & Hightsoe, 2003 | 3 | 100 | Children | 100 | General education | Recess | Antecedent-based | 5.28 |
| Rock, 2005 | 2 | 100 | Children | 50 | General education | Strategic self-monitoring | Self-regulation | 6.37 |
| Rooney, Hallahan, & Lloyd, 1984 | 4 | 50 | Children | - | General education | Self-recording | Self-regulation | 7.00  (Winsorized) |
| Rooney, Polloway, & Hallahan, 1985 | 3 | 100 | Children | - | Other | Self-monitoring | Self-regulation | 2.16 |
| Schilling, Washington, Billingsley, & Deitz, 2003 | 3 | 67 | Children | 100 | General education | Therapy balls | Antecedent-based | 4.39 |
| Shimabukuro, Prater, Jenkins, & Edelen-Smith, 1999 | 3 | 100 | Adolescents | - | Other | Self-monitoring | Self-regulation | 4.80 |
| Skinner, Veerkamp, Kamps, & Andra, 2009 | 1 | 100 | Children | 100 | General education | Fixed-time reinforcement | Consequence-based | 4.30 |
| Stahr, Cushing, Lane, & Fox, 2006 | 1 | 100 | Children | 100 | Other | Function-based intervention | Combined | 2.61 |
| Stewart & McLaughlin, 1992 | 1 | 100 | Adolescents | - | Other | Self-recording | Self-regulation | 5.29 |
| Swenson, Lolich, Williams, & McLaughlin, 2000 | 1 | 100 | Adolescents | - | Other | Structured free-time | Consequence-based | 2.33 |
| Waller, Albertini, & Waller, 2011^1^ | 1 | 0 | Children | - | Other | Self-monitoring | Self-regulation | 3.07 |
| Waller, Albertini, & Waller, 2011^2^ | 1 | 0 | Children | - | Other | Self-monitoring + antecedent teacher attention + reinforcement | Combined | 4.70 |
| Williamson, Calpin, DiLorenzo, Garris, & Petti, 1981 | 1 | 100 | Children | 100 | - | Instructions + guided practice | Antecedent-based | 0.42 |

*Note.* med = medicated.

^a^Superscript numbers are added to references that yielded more than one study.
