# Supplementary material for: The Effects of Classroom Interventions on Off-Task and Disruptive Classroom Behavior in Children with Symptoms of Attention-Deficit/Hyperactivity Disorder: A Meta-Analytic Review
Source: PLoS One. 2016 Feb 17;11(2):e0148841. doi: 10.1371/journal.pone.0148841 (PMC4757442; doi:10.1371/journal.pone.0148841)
Supplement: S1 Text — (DOCX) [file pone.0148841.s006.docx]

**S1 Text. Computation of Effect Sizes.**

Effect sizes used for WSD studies were based on the standardized mean-change measure outlined by Becker (1988). For both the intervention and control group, standardized mean changes were computed by dividing the difference between the means of the posttest and pretest assessment by the pooled standard deviation. For studies not using a control group, values were imputed of a fictive control group of the same size as the intervention group and with a standardized mean change of zero. The standardized mean changes were then corrected for small sample sizes, resulting in an unbiased estimator (Becker, 1988; Hedges, 1981). Finally, the unbiased standardized mean change of the control group was subtracted from that of the intervention group to generate the effect size used in the meta-analysis. The majority of studies did not provide sufficient information to calculate the correlation between the pretest and posttest measures. Therefore, a conservative value of .30 was imputed. The exact formula used for computation of effect sizes and variances for WSD studies are

$g_{\mathrm{ij}}=\left[ 1- \frac{3}{4\left( n_{\mathrm{ij}}- 1 \right) - 1} \right] \frac{\left( \bar{Y}_{\mathrm{ij}}- \bar{X}_{\mathrm{ij}} \right)}{S_{\mathrm{ij}}}$ with v$ar(g_{\mathrm{ij}})=\frac{2\left( 1 -r_{\mathrm{ij}} \right)}{n_{\mathrm{ij}}}+\frac{{g_{\mathrm{ij}}}^{2}}{2n_{\mathrm{ij}}}$

${SMD}_{i}=g_{i1}-g_{i2}$ with $\mathrm{var}\left( {SMD}_{i} \right)=var(g_{i1})+var\left( g_{i2} \right)$

where $\bar{X}$_ij_ is the group pretest mean, $\bar{Y}$_ij_ is the group posttest mean, *S*_ij_ is the pooled standard deviation, and *r*_ij_ is the correlation between pretest and posttest scores for group j in study i.

References

Becker, B. J. (1988). Synthesizing standardized mean-change measures. *British Journal of Mathematical and Statistical Psychology, 41*(2), 257-278.

Hedges, L. V. (1981). Distribution theory for glass's estimator of effect size and related estimators. *Journal of Educational Statistics, 6*(2), 107-128.
