# Supplementary material for: The Effects of Classroom Interventions on Off-Task and Disruptive Classroom Behavior in Children with Symptoms of Attention-Deficit/Hyperactivity Disorder: A Meta-Analytic Review
Source: PLoS One. 2016 Feb 17;11(2):e0148841. doi: 10.1371/journal.pone.0148841 (PMC4757442; doi:10.1371/journal.pone.0148841)
Supplement: S2 Text — (DOCX) [file pone.0148841.s007.docx]

**S2 Text. List of Studies Included in the Meta-Analytic Review.**

Abramowitz, A. J., Eckstrand, D., O'Leary, S. G., & Dulcan, M. K. (1992). ADHD children's responses to stimulant medication and two intensities of a behavioral intervention. *Behavior Modification, 16*(2), 193-203. doi:10.1177/01454455920162003

Alter, P. J., Wyrick, A., Brown, E. T., & Lingo, A. (2008). Improving mathematics problem solving skills for students with challenging behavior. *Beyond Behavior, 17*(3), 2-7.

Anhalt, K., McNeil, C., & Bahl, A. (1998). The ADHD classroom kit: A whole-classroom approach for managing disruptive behavior. *Psychology in the Schools, 35*(1), 67-79. doi:10.1002/(SICI)1520-6807(199801)35:1<67::AID-PITS6>3.0.CO;2-R

Ardoin, S. P., & Martens, B. K. (2004). Training children to make accurate self-evaluations: Effects on behavior and the quality of self-ratings. *Journal of Behavioral Education, 13*(1), 1-23. doi:10.1023/B:JOBE.0000011257.63085.88

Banda, D. R., & Sokolosky, S. (2012). Effectiveness of noncontingent attention to decrease attention-maintained disruptive behaviors in the general education classroom. *Child & Family Behavior Therapy, 34*(2), 130-140. doi:10.1080/07317107.2012.684646

Barkley, R., Copeland, A., & Sivage, C. (1980). A self-control classroom for hyperactive-children. *Journal of Autism and Developmental Disorders, 10*(1), 75-89. doi:10.1007/BF02408435

Barry, L., & Messer, J. (2003). A practical application of self-management for students diagnosed with attention-deficit/hyperactivity disorder. *Journal of Positive Behavior Interventions, 5*(4), 238-248. doi:10.1177/10983007030050040701

Bloomquist, M. L., August, G. J., & Ostrander, R. (1991). Effects of a school-based cognitive-behavioral intervention for ADHD children. *Journal of Abnormal Child Psychology, 19*(5), 591-605. doi:10.1007/BF00925822

Bowers, D. S., Clement, P. W., Fantuzzo, J. W., & Sorensen, D. A. (1985). Effects of teacher-administered and self-administered reinforcers on learning disabled children. *Behavior Therapy, 16*(4), 357-369. doi:10.1016/S0005-7894(85)80003-4

Broussard, C. D., & Northup, J. (1995). An approach to functional assessment and analysis of disruptive behavior in regular education classrooms. *School Psychology Quarterly, 10*(2), 151-164. doi:10.1037/h0088301

Broussard, C., & Northup, J. (1997). The use of functional analysis to develop peer interventions for disruptive classroom behavior. *School Psychology Quarterly, 12*(1), 65-76.

Burley, R., & Waller, R. J. (2005). Effects of a collaborative behavior management plan on reducing disruptive behaviors of a student with ADHD. *TEACHING Exceptional Children Plus, 1*(4).

Cameron, M. I., & Robinson, V. M. J. (1980). Effects of cognitive training on academic and on-task behavior of hyperactive children. *Journal of Abnormal Child Psychology, 8*(3), 405-19.

Campbell, A., & Anderson, C. M. (2011). Check-In/check-out: A systematic evaluation and component analysis. *Journal of Applied Behavior Analysis, 44*(2), 315-326. doi:10.1901/jaba.2011.44-315

Carter, D. R., & Horner, R. H. (2009). Adding function-based behavioral support to first step to success integrating individualized and manualized practices. *Journal of Positive Behavior Interventions, 11*(1), 22-34. doi:10.1177/1098300708319125

Christie, D. J., Hiss, M., & Lozanoff, B. (1984). Modification of inattentive classroom behavior: Hyperactive children's use of self-recording with teacher guidance. *Behavior Modification, 8*(3), 391-406. doi:10.1177/01454455840083006

Coleman, R. (1970). A conditioning technique applicable to elementary school classrooms. *Journal of Applied Behavior Analysis, 3*(4), 293-297.

Davies, S., & Witte, R. (2000). Self-management and peer-monitoring within a group contingency to decrease uncontrolled verbalizations of children with attention-deficit/hyperactivity disorder. *Psychology in the Schools, 37*(2), 135-147. doi:10.1002/(SICI)1520-6807(200003)37:2<135::AID-PITS5>3.0.CO;2-U

DiGangi, S. A., Maag, J. W., & Rutherford, R. B. (1991). Self-graphing of on-task behavior: Enhancing the reactive effects of self-monitoring on on-task behavior and academic performance. *Learning Disability Quarterly, 14*(3), 221-230.

DiGennaro, F. D., Martens, B. K., & McIntyre, L. L. (2005). Increasing treatment integrity through negative reinforcement: Effects on teacher and student behavior. *School Psychology Review, 34*(2), 220-231.

Ducharme, J. M., & Harris, K. E. (2005). Errorless embedding for children with on-task and conduct difficulties: Rapport-based, success-focused intervention in the classroom. *Behavior Therapy, 36*(3), 213-222. doi:10.1016/S0005-7894(05)80070-X

Dunlap, G., dePerczel, M., Clarke, S., Wilson, D., Wright, S., White, R., & Gomez, A. (1994). Choice making to promote adaptive behavior for students with emotional and behavioral challenges. *Journal of Applied Behavior Analysis, 27*(3), 505-518. doi:10.1901/jaba.1994.27-505

DuPaul, G. J., Ervin, R. A., Hook, C. L., & McGoey, K. E. (1998). Peer tutoring for children with attention deficit hyperactivity disorder: Effects on classroom behavior and academic performance. *Journal of Applied Behavior Analysis, 31*(4), 579-592. doi:10.1901/jaba.1998.31-579

DuPaul, G. J., Guevremont, D. C., & Barkley, R. A. (1992). Behavioral treatment of attention-deficit hyperactivity disorder in the classroom: The use of the attention training system. *Behavior Modification, 16*(2), 204-225. doi:10.1177/01454455920162004

DuPaul, G. J., & Henningson, P. N. (1993). Peer tutoring effects on the classroom performance of children with attention deficit hyperactivity disorder. *School Psychology Review, 22*(1), 134-143.

Eastman, B., & Rasbury, W. (1981). Cognitive self-instruction for the control of impulsive classroom-behavior - ensuring the treatment package. *Journal of Abnormal Child Psychology, 9*(3), 381-387. doi:10.1007/BF00916842

Ervin, R. A., DuPaul, G. J., Kern, L., & Friman, P. C. (1998). Classroom-based functional and adjunctive assessments: Proactive approaches to intervention selection for adolescents with attention deficit hyperactivity disorder. *Journal of Applied Behavior Analysis, 31*(1), 65-78. doi:10.1901/jaba.1998.31-65

Evans, S. W., & Others, A. (1995). The efficacy of notetaking to improve behavior and comprehension of adolescents with attention deficit hyperactivity disorder. *Exceptionality, 5*(1), 1-17.

Fabiano, G., & Pelham, W. (2003). Improving the effectiveness of behavioral classroom interventions for attention-deficit/hyperactivity disorder: A case study. *Journal of Emotional and Behavioral Disorders, 11*(2), 122-128. doi:10.1177/106342660301100206

Fedewa, A. L., & Erwin, H. E. (2011). Stability balls and students with attention and hyperactivity concerns: Implications for on-task and in-seat behavior. *American Journal of Occupational Therapy, 65*(4), 393-399. doi:10.5014/ajot.2011.000554

Flynn, N. M., & Rapoport, J. L. (1976). Hyperactivity in open and traditional classroom environments. *The Journal of Special Education, 10*(3), 285-290. doi:10.1177/002246697601000309

Germer, K. A., Kaplan, L. M., Giroux, L. N., Markham, E. H., Ferris, G. J., Oakes, W. P., & Lane, K. L. (2011). A function-based intervention to increase a second-grade student's on-task behavior in a general education classroom. *Beyond Behavior, 20*(3), 19-30.

Gordon, M., & Others, A. (1991). Nonmedical treatment of ADHD/Hyperactivity: The attention training system. *Journal of School Psychology, 29*(2), 151-59.

Graham-Day, K., Gardner, R. I.,II, & Hsin, Y. (2010). Increasing on-task behaviors of high school students with attention deficit hyperactivity disorder: Is it enough? *Education & Treatment of Children, 33*(2), 205-221. doi:10.1353/etc.0.0096

Guderjahn, L., Gold, A., Stadler, G., & Gawrilow, C. (2013). Self-regulation strategies support children with ADHD to overcome symptom-related behavior in the classroom. *ADHD Attention Deficit and Hyperactivity Disorders,* , 1-11. doi:10.1007/s12402-013-0117-7

Gureasko-Moore, S., DuPaul, G. J., & White, G. P. (2006). The effects of self-management in general education classrooms on the organizational skills of adolescents with ADHD. *Behavior Modification, 30*(2), 159-183. doi:10.1177/0145445503259387

Gureasko-Moore, S., DuPaul, G. J., & White, G. P. (2007). Self-management of classroom preparedness and homework: Effects on school functioning of adolescents with attention deficit hyperactivity disorder. *School Psychology Review, 36*(4), 647-664.

Hallahan, D. P., Lloyd, J. W., Kneedler, R. D., & Marshall, K. J. (1982). A comparison of the effects of self- versus teacher-assessment of on-task behavior. *Behavior Therapy, 13*(5), 715-723. doi:10.1016/S0005-7894(82)80027-0

Hallahan, D. P., Lloyd, J., Kosiewicz, M. M., Kauffman, J. M., & Graves, A. W. (1979). Self-monitoring of attention as a treatment for a learning disabled boy's off-task behavior. *Learning Disability Quarterly, 2*(3), 24-32.

Harris, K. R. (1986). Self-monitoring of attentional behavior versus self-monitoring of productivity: Effects on on-task behavior and academic response rate among learning disabled children. *Journal of Applied Behavior Analysis, 19*(4), 417-23.

Harris, K. R., Friedlander, B. D., Saddler, B., Frizzelle, R., & Graham, S. (2005). Self-monitoring of attention versus self-monitoring of academic performance: Effects among students with ADHD in the general education classroom. *The Journal of Special Education, 39*(3), 145-156. doi:10.1177/00224669050390030201

Hart, K. C., Massetti, G. M., Fabiano, G. A., Pariseau, M. E., & Pelham, W. E., Jr. (2011). Impact of group size on classroom on-task behavior and work productivity in children with ADHD. *Journal of Emotional and Behavioral Disorders, 19*(1), 55-64. doi:10.1177/1063426609353762

Hoff, K. E., & Ervin, R. A. (2013). Extending self-management strategies: The use of a classwide approach. *Psychology in the Schools, 50*(2), 151-164. doi:10.1002/pits.21666

Hoff, K. E., Ervin, R. A., & Friman, P. C. (2005). Refining functional behavioral assessment: Analyzing the separate and combined effects of hypothesized controlling variables during ongoing classroom routines. *School Psychology Review, 34*(1), 45-57.

Horn, W. F., Chatoor, I., & Conners, C. K. (1983). Additive effects of dexedrine and self-control training: A multiple assessment. *Behavior Modification, 7*(3), 383-402. doi:10.1177/01454455830073006

Iskander, J. M., & Rosales, R. (2013). An evaluation of the components of a social stories™ intervention package. *Research in Autism Spectrum Disorders, 7*(1), 1-8. doi:10.1016/j.rasd.2012.06.004

Jacob, R. G., O'Leary, K. D., & Rosenblad, C. (1978). Formal and informal classroom settings: Effects on hyperactivity. *Journal of Abnormal Child Psychology, 6*(1), 47-59. doi:10.1007/BF00915781

Jurbergs, N., Palcic, J. L., & Kelley, M. L. (2010). Daily behavior report cards with and without home-based consequences: Improving classroom behavior in low income, african american children with ADHD. *Child & Family Behavior Therapy, 32*(3), 177-195. doi:10.1080/07317107.2010.500501

Kapalka, G. M. (2005). Avoiding repetitions reduces ADHD children's management problems in the classroom. *Emotional & Behavioural Difficulties, 10*(4), 269-279. doi:10.1177/1363275205058999

Kern, L., Delaney, B., Clarke, S., Dunlap, G., & Childs, K. (2001). Improving the classroom behavior of students with emotional and behavioral disorders using individualized curricular modifications. *Journal of Emotional and Behavioral Disorders, 9*(4), 239-247. doi:10.1177/106342660100900404

Kubany, E. S., Weiss, L. E., & Sloggett, B. B. (1971). The good behavior clock: A reinforcement/time out procedure for reducing disruptive classroom behavior. *Journal of Behavior Therapy and Experimental Psychiatry, 2*(3), 173-179. doi:10.1016/0005-7916(71)90055-3

Lee, Y., Sugai, G., & Horner, R. H. (1999). Using an instructional intervention to reduce problem and off-task behaviors. *Journal of Positive Behavior Interventions, 1*(4), 195-204. doi:10.1177/109830079900100402

Lloyd, J. W., Hallahan, D. P., Kosiewicz, M. M., & Kneedler, R. D. (1982). Reactive effects of self-assessment and self-recording on attention to task and academic productivity. *Learning Disability Quarterly, 5*(3), 216-227.

Lo, Y., & Cartledge, G. (2006). FBA and BIP: Increasing the behavior adjustment of african american boys in schools. *Behavioral Disorders, 31*(2), 147-161.

Locke, W. R. F.,Lynn S. (1995). Effects of peer-mediated reading instruction on the on-task behavior and social interaction of.. *Journal of Emotional & Behavioral Disorders, 3*(2), 92.

Maag, J. W., Rutherford, R. B., & DiGangi, S. A. (1992). Effects of self-monitoring and contingent reinforcement on on-task behavior and academic productivity of learning-disabled students: A social validation study. *Psychology in the Schools, 29*(2), 157-172.

Majeika, C. E., Walder, J. P., Hubbard, J. P., Steeb, K. M., Ferris, G. J., Oakes, W. P., & Lane, K. L. (2011). Improving on-task behavior using a functional assessment-based intervention in an inclusive high school setting. *Beyond Behavior, 20*(3), 55-66.

Mathes, M. Y., & Bender, W. N. (1997). The effects of self-monitoring on children with attention deficit/hyperactivity disorder who are receiving pharmacological interventions. *Remedial and Special Education, 18*(2), 121-128. doi:10.1177/074193259701800206

Mautone, J. A., DuPaul, G. J., & Jitendra, A. K. (2005). The effects of computer-assisted instruction on the mathematics performance and classroom behavior of children with ADHD. *Journal of Attention Disorders, 9*(1), 301-312. doi:10.1177/1087054705278832

Miranda, A., Presentacion, M., & Soriano, M. (2002). Effectiveness of a school-based multicomponent program for the treatment of children with ADHD. *Journal of Learning Disabilities, 35*(6), 546-562.

Miranda, A., Jarque, S., & Rosel, J. (2006). Treatment of children with ADHD: Psychopedagogical program at school versus psychostimulant medication. *Psicothema, 18*(3), 335-341.

Nolan, J. D., & Filter, K. J. (2012). A function-based classroom behavior intervention using non-contingent reinforcement plus response cost. *Education & Treatment of Children, 35*(3), 419-430. doi:10.1353/etc.2012.0017

Northup, J., Broussard, C., Jones, K., & George, T. (1995). The differential effects of teacher and peer attention on the disruptive classroom behavior of three children with a diagnosis of attention deficit hyperactivity disorder. *Journal of Applied Behavior Analysis, 28*(2), 227-228. doi:10.1901/jaba.1995.28-227

Ota, K. R., & DuPaul, G. J. (2002). Task engagement and mathematics performance in children with attention-deficit hyperactivity disorder: Effects of supplemental computer instruction. *School Psychology Quarterly, 17*(3), 242-257. doi:10.1521/scpq.17.3.242.20881

Palcic, J. L., Jurbergs, N., & Kelley, M. L. (2009). A comparison of teacher and parent delivered consequences: Improving classroom behavior in low-income children with ADHD. *Child & Family Behavior Therapy, 31*(2), 117-133. doi:10.1080/07317100902910513

Pang, W. C., & Zhang, K. C. (2011). Reading intervention for secondary students with hyperactive behaviours in hong kong. *Emotional & Behavioural Difficulties, 16*(1), 69-85.

Paniagua, F. A., Morrison, P. B., & Black, S. A. (1990). Management of a hyperactive-conduct disordered child through correspondence training: A preliminary study. *Journal of Behavior Therapy and Experimental Psychiatry, 21*(1), 63-68. doi:10.1016/0005-7916(90)90050-U

Paniagua, F. A., Pumariega, A. J., & Black, S. A. (1988). Clinical effects of correspondence training in the management of hyperactive children. *Behavioral Residential Treatment, 3*(1), 19-40. doi:10.1002/bin.2360030103

Pariseau, M. E., Fabiano, G. A., Massetti, G. M., Hart, K. C., & Pelham, W. E., Jr. (2010). Extended time on academic assignments: Does increased time lead to improved performance for children with attention-Deficit/Hyperactivity disorder? *School Psychology Quarterly, 25*(4), 236-248. doi:10.1037/a0022045

Pelham, W. E., Waschbusch, D. A., Hoza, B., Gnagy, E. M., Greiner, A. R., Sams, S. E., . . . Carter, R. L. (2011). Music and video as distractors for boys with ADHD in the classroom: Comparison with controls, individual differences, and medication effects. *Journal of Abnormal Child Psychology, 39*(8), 1085-1098.

Pfiffner, L. J., Rosén, L. A., & O'Leary, S. G. (1985). The efficacy of an all-positive approach to classroom management. *Journal of Applied Behavior Analysis, 18*(3), 257-261. doi:10.1901/jaba.1985.18-257

Powell, S., & Nelson, B. (1997). Effects of choosing academic assignments on a student with attention deficit hyperactivity disorder. *Journal of Applied Behavior Analysis, 30*(1), 181-183. doi:10.1901/jaba.1997.30-181

Price, A. T., Martella, R. C., Marchand-Martella, N., & Cleanthous, C. C. (2002). A comparison of immediate feedback delivered via an FM headset versus delayed feedback on the inappropriate verbalizatins of a student with ADHD. *Education & Treatment of Children, 25*(2), 159-171.

Rafferty, L. A., Arroyo, J., Ginnane, S., & Wilczynski, K. (2011). Self-monitoring during spelling practice: Effects on spelling accuracy and on-task behavior of three students diagnosed with attention deficit hyperactivity disorder. *Behavior Analysis in Practice, 4*(1), 37-45.

Rapport, M. D., Murphy, H. A., & Bailey, J. S. (1982). Ritalin vs. response cost in the control of hyperactive children: A within-subject comparison. *Journal of Applied Behavior Analysis, 15*(2), 205-216. doi:10.1901/jaba.1982.15-205

Rapport, M. D., & Others, A. (1980). The effects of a response cost treatment tactic on hyperactive children. *Journal of School Psychology, 18*(2), 98-111.

Ridgway, A., Northup, J., Pellegrin, A., LaRue, R., & Hightsoe, A. (2003). Effects of recess on the classroom behavior of children with and without attention-deficit hyperactivity disorder. *School Psychology Quarterly, 18*(3), 253-268. doi:10.1521/scpq.18.3.253.22578

Rock, M. L. (2005). Use of strategic self-monitoring to enhance academic engagement, productivity, and accuracy of students with and without exceptionalities. *Journal of Positive Behavior Interventions, 7*(1), 3-17.

Rooney, K. J., Hallahan, D. P., & Lloyd, J. W. (1984). Self-recording of attention by learning disabled students in the regular classroom. *Journal of Learning Disabilities, 17*(6), 360-364.

Rooney, K., Polloway, E. A., & Hallahan, D. P. (1985). The use of self-monitoring procedures with low IQ learning disabled students. *Journal of Learning Disabilities, 18*(7), 384-389. doi:10.1177/002221948501800703

Rosén, L. A., O'Leary, S. G., Joyce, S. A., Conway, G., & Pfiffner, L. J. (1984). The importance of prudent negative consequences for maintaining the appropriate behavior of hyperactive students. *Journal of Abnormal Child Psychology, 12*(4), 581-604. doi:10.1007/BF00916852

Schilling, D. L., Washington, K., Billingsley, F. F., & Deitz, J. (2003). Classroom seating for children with attention deficit hyperactivity disorder: Therapy balls versus chairs. *American Journal of Occupational Therapy, 57*(5), 534-541. doi:10.5014/ajot.57.5.534

Shimabukuro, S. M., Prater, M. A., Jenkins, A., & Edelen-Smith, P. (1999). The effects of self-monitoring of academic performance on students with learning disabilities and ADD/ADHD. *Education & Treatment of Children, 22*(4), 397-414.

Skinner, J. N., Veerkamp, M. B., Kamps, D. M., & Andra, P. R. (2009). Teacher and peer participation in functional analysis and intervention for a first grade student with attention deficit hyperactivity disorder. *Education & Treatment of Children, 32*(2), 243-266. doi:10.1353/etc.0.0059

Stahr, B., Cushing, D., Lane, K., & Fox, J. (2006). Efficacy of a function-based intervention in decreasing off-task behavior exhibited by a student with ADHD. *Journal of Positive Behavior Interventions, 8*(4), 201-211. doi:10.1177/10983007060080040301

Stewart, K. G., & McLaughlin, T. F. (1992). Self-recording: Effects of reducing off-task behavior with a high school student with an attention deficit hyperactivity disorder. *Child & Family Behavior Therapy, 14*(3), 53-59. doi:10.1300/J019v14n03_04

Swenson, N., Lolich, E., Williams, R. L., & McLaughlin, T. F. (2000). The effects of structured free-time on request compliance and on-task behavior of a preadolescent with ADHD. *Child & Family Behavior Therapy, 22*(1), 51-59. doi:10.1300/J019v22n01_04

Waller, R. J., Albertini, C. L., & Waller, K. S. (2011). Self-monitoring of performance to promote accurate work completion: A functional based intervention for a 4th grade student presenting challenging behavior. *Advances in School Mental Health Promotion, 4*(1), 52-60. doi:10.1080/1754730X.2011.9715623

Williamson, D. A., Calpin, J. P., DiLorenzo, T. M., Garris, R. P., & Petti, T. A. (1981). Treating hyperactivity with dexedrine and activity feedback. *Behavior Modification, 5*(3), 399-416. doi:10.1177/014544558153008
